# Epidemiology and associated factors of depression among cancer patients in Ethiopia: protocol for systematic review and meta-analysis

**DOI:** 10.1186/s13033-022-00556-5

**Published:** 2022-09-02

**Authors:** Mekonnen Tsehay, Asmare Belete, Mogesie Necho

**Affiliations:** College of Medicine and Health Sciences, Department of Psychiatry, Wollo University, Dessie, Ethiopia

**Keywords:** Cancer, Depression

## Abstract

**Background:**

There is no pooled evidence regarding the prevalence and potential associated factors of depression among cancer patients in Ethiopian community. Hence, the current review aimed to examine the prevalence and associated factors of depression among cancer patients in Ethiopia.

**Method:**

A computerized systematic literature search was made in MEDLINE, Scopus, PubMed, Science Direct, and Google Scholar. Each database was searched from its start date to June 2020. More over we will also add scholars and gray literature consultations. All articles will be included if they were published in English, which evaluated the prevalence and associated factors of depression among cancer patients in Ethiopia. Pooled estimations with a 95% confidence interval (CI) were calculated with DerSimonian-Laird random-effects model. Publication bias was evaluated by using inspection of funnel plots and statistical tests.

**Discussion:**

Since we are using existing anonymized data, ethical approval is not required for this study. Our results can be used to guide clinical decisions about the most efficient way to prevent and treat depression among cancer patients.

*Systematic review registration* Submitted to Prospero.

## Introduction

There were 17 million new cases of cancer worldwide [1]. The four most common cancers occurring worldwide are lung, female breast, bowel, and prostate cancer. Lung, liver, stomach, and bowel are the most common causes of cancer death worldwide, accounting for more than four in ten of all cancer deaths [1].

A person’s risk of developing cancer depends on many factors, including age, genetics, and exposure to risk factors (including some potentially avoidable lifestyle factors). Cancer risk factors are overall similar worldwide. Smoking, insufficient physical activity, alcohol, diet, overweight and obesity, and infections account for a high proportion of cancers worldwide [2, 3].

Cancer is a life-threatening and feared diagnosis, and is a source of great distress in patients. One in three people with cancer will experience a mental health problem such as depression, or anxiety disorders before, during or after treatment [4].

A cancer diagnosis, its associated symptoms, and treatment can have a significant emotional impact on people and their families, with fear, isolation, loss of self-esteem, and loss of independence having an impact [5].

Everyone knows it is better to catch cancer earlier, at stage one instead of later at stage four [6]. The same is true for mental health conditions. Unfortunately, many people with cancer are never told about the chance they will develop a mental health condition like depression nor will they receive treatment for it [7].

It is estimated that up to one-third of people treated for cancer in hospitals have a common mental health condition [8]. Rates of major depressive disorder are thought to be up to three times higher than in the general population [9]. Anywhere from 8 to 24% of people with cancer are also livings with depression [10]. Youth and young adults are at greater risk for depression and other conditions compared to adults with cancer [11].

However mental health problems that arise as a result of cancer are too often sidelined, and there are several reasons why a person with cancer may not get help for their mental health condition: Cancer, depression and anxiety have shared symptoms like fatigue, lack of sleep, and decreased appetite which can make recognizing mental health conditions difficult [12–14]. This is a group that regularly faces threats to life and figuring out what is a regular reaction to cancer diagnosis and treatment versus signs one has a mental health condition can be hard.

Cancer care teams often lack specific skills to recognize mental health conditions. Some in the community do not agree on what depression is and looks like. With so much time and money spent on cancer treatment, many are forced to see their mental health as less important and do not seek help [14]. For example, they may be less likely to exercise, more likely to drink too much alcohol or miss therapy appointments.

Depression affects the quality of life (QOL) and compromises patient outcomes, with depression resulting in higher rates of mortality in cancer [15]. A meta‑analysis revealed that minor or major depression increases mortality rates by 39% [16].

People, who get treatment, often see improvement in their overall medical condition, are more likely to follow through with medical care, and have a better quality of life. The study found that those who got treatment and had fewer symptoms of depression had longer average survival times than those who had more symptoms [17].

The prevalence of depression in cancer patients is thought to be up to three times higher than in the general population [18]. Studies have identified a variety of prevalence ranging from 2.0 to 43.5% [19, 20], whilst palliative care wards have documented rates of depression as high as 49.0% [21]. The wide range of reported prevalence may be due to differences in assessment tools, variation in the types of patients interviewed, and varying age groups, varying gender proportions, inpatient status, and other factors. Knowledge of having cancer and stage of the disease were also significantly associated with the occurrence of depression [22].

Even though multiple studies have been performed and showed that cancer patients have extensive variations in the prevalence and associated factors of depression across different communities of sub-Saharan Africa, there is no pooled evidence regarding the overall prevalence and potential associated factors of depression among cancer patients. The objective of the current review is to present an overview of the magnitude and associated factors of depression among cancer patients in Ethiopia. Knowledge on the pooled magnitude of depression and detecting its determinants would assist policy-makers and program implementers in deciding evidence-driven prevention and promotion and treatment activities in this area. So, this systematic review and meta-analysis study is intended to review the existing pieces of evidence on the prevalence of depression and its risk factors and present the pooled prevalence of synergized effect in cancer patients in Ethiopia.

## Methods

### Data sources and search strategies

Articles for review will be searched from the 5 databases: MEDLINE, Scopus, PubMed, ScienceDirect, and Google Scholar. And also scholars and gray literature consultations will be used. Each database will be searched from its start date to october 2022 by using the following words: key terms and words: (“(Prevalence OR epidemiology OR magnitude OR incidence) AND (“depression” OR “depression disorders” OR “depression symptoms”) AND (cancer) AND (factor OR risk OR (“risk factor” OR determinant) AND (Ethiopia OR Eritrea OR Kenya OR Uganda OR Tanzania OR Sudan OR Djibouti OR Somalia OR Rwanda OR “east Africa”)”). The literature search will be conducted by two separate researchers (Mekonnen Tsehay and Moges Necho) to avoid missing articles.

### Study selection and eligibility

#### Study selection

All duplicated searches will be removed using the ENDNOTE software version X5 (Thomson Reuters, USA). Two authors (Asmare Belete and Mekonnen Tsehay) screened the titles and abstracts of identified articles by applying the inclusion criteria. Two authors (Mekonnen tsehay and Asmare Belete) independently will review the full text. The final inclusion of the studies will be determined by agreement of both reviewers and when there is disagreement, a third author (Mogese Necho) will be involved. All the authors will be involved in the discussion and agreed on the final inclusion.

#### Inclusion criteria and quality assessment

The PRISMA guidelines protocol will be used to write the systematic review [23]. Eligibility criteria will be defined as follows: (1) Cross-sectional, cohort and case–control study design, (2) Subject of study should be any type of cancer patients in each study, (3) Research articles should be published in the English language, (4) Articles which reports prevalence of depression in cancer patients [24] Articles identifies risk factors of depression in cancer patients, and (6) Studies should be done in Ethiopia. And (1) we will exclude letters, reviews, interventional studies, commentaries, and editorials, and (2) Studies duplicated will also be excluded before being doubled in the analysis.

Two review authors (Mekonnen Tsehay and Moges Necho) will assess the quality of all included studies independently. Discrepancies between these review authors will be resolved by a third reviewer as necessary. The modified version of Newcastle–Ottawa Quality Assessment tool (Stang A. Critical evaluation of the Newcastle–Ottawa scale for the assessment of the quality of nonrandomized studies in meta-analyses will be used as guideline for quality appraisal of included studies [25]. Representativeness and size of the sample, comparability between study subjects, ascertainment of depression symptoms, and statistical quality were the dimensions of the Newcastle–Ottawa scale in assessing the quality of each study. Quantitatively, quality scores of each study will be obtained by dividing the score of each study to the highest scoring study from the included studies.

### Data extraction and statistical analysis

Data on study design, year of study, and study setting, type of cancer, cancer treatment phase, and prevalence of depression will be retrieved. Data about the prevalence of depression among cancer patients and possible associated factors will be extracted from the eligible articles. Data will be extracted by two researchers (Mekonnen Tsehay and Moges Necho) and cross-checked to minimize error. Data will be entered into Comprehensive Meta-Analysis version 20 and analysis will be carried out to determine the pooled prevalence of depression among cancer patients and relative risk of the associated factors by using a random-effects model to combine results of included studies in the meta-analysis. The heterogeneity in pooled estimation will be determined by using (DL-method) [26]. Sensitivity analysis will be also carried out to detect any sources of variation in the pooled estimation. Moreover, publication bias will be evaluated by inspection of funnel plots and using Egger and Begg’s tests.

## Discussion

This meta-analysis will meet the criteria for waiver of ethical approval as defined by McMaster University Ethics Board as it will use existing anonymized data. The prognostic factors that will be identified from our meta-analysis will help to inform the development of a more sophisticated risk prediction model for assessing factors of depression in cancer patients.

## Conclusion

This will ultimately be used to guide clinical decisions about the most efficient way to prevent and treat depression among cancer patients. We will publish the results of our study in an open-access scientific journal and will disseminate our findings in relevant national and international conferences.

## Data Availability

On reasonable request or as the supplementary file will be uploaded.

## Abbreviations

CI: Confidence interval
CES-D: Center for Epidemiology Studies Depression Scale
DALYs: Disability Adjusted Life Years
DSM-V-TR: Diagnostic and Statistical Manual of Mental Disorders Fifth Edition-Text Revision
E.C: Ethiopian Calendar
G.C: Gregorian Calendar
PHC: Primary Health Care
PHQ 9: Patient Health Questionnaire-9
PPS: Population Proportion to Size
SPSS: Statistical Package for Social Sciences
WHO: World Health Organization
